# Wide excision and reconstruction surgery for recurrent sweat gland umbilical adenocarcinoma followed by chemotherapy can prevent the risk of recurrences

**DOI:** 10.1186/s12893-018-0421-4

**Published:** 2018-10-12

**Authors:** Adeodatus Yuda Handaya, Nova Yuli Prasetyo Budi, Guntur Marganing Adi Nugroho, Aditya Rifqi Fauzi

**Affiliations:** 1 Digestive Surgery Division, Department of Surgery, Faculty of Medicine, Universitas Gadjah Mada/Dr. Sardjito Hospital, Jl. Kesehatan No. 1, Yogyakarta, 55281 Indonesia; 2 Faculty of Medicine, Universitas Gadjah Mada/Dr. Sardjito Hospital, Yogyakarta, 55281 Indonesia

**Keywords:** Umbilical tumor, Sudoriferous adenocarcinoma, Cancer recurrence, Radical excision, Reconstruction

## Abstract

**Background:**

Adenocarcinoma derived from umbilicus is very rare. Most adenocarcinomas in umbilicus are secondary events. Carcinoma derived from sweat glands is sporadic, highly radioresistant and has a clinical appearance that is difficult to predict.

**Case presentation:**

A 37-year-old woman presented with recurrent umbilicus adenocarcinoma after a history of umbilicus tumor surgery 14 months earlier and Capecitabine chemotherapy six times. Malignant cells were found in Fine Needle Aspiration Biopsy (FNAB) examination. A colonoscopy examination found pathological colitis without any colonic mass. The patient underwent wide excision and reconstruction surgery using a composite attachment visceral mesh with a size of 30 × 30 cm. Histopathologic examination of the surgery diagnosed adenocarcinoma of the sudoriferous gland with adjacent tissue free of tumor cells. Six months post operation, Positron Emission Tomography (PET) scan was performed and found neither residue nor recurrence.

**Conclusions:**

Wide excision and reconstruction surgery for recurrent sweat gland umbilical adenocarcinoma followed by chemotherapy can be an alternative to prevent recurrences.

## Background

Primary umbilical tumors are a sporadic case, with only a few cases being reported in today’s era of modern medicine. Although rarely found, umbilical tumors events ranging from one-sixth to one-fourth of all malignancy events in this location. Metastasis of the umbilicus tumor is more common. Umbilical adenocarcinoma primary tumors can grow from different tissues, from pre-existing endometriomas, coelomic mesothelium or the embryological remains of the umbilicus, both from the vitello-intestinal (omphalo-mesenteric) and urachus tracts [1, 2].

Sweat gland carcinoma has local destruction capability and local tissue infiltration, and also distant metastasis which primarily occurs in adult patients with peak incidence in the fifth and sixth decades of life. The predilection of this tumor is in the genital skin and perineum (34.5%), trunk (26.4%), head and neck (18.3%) and lower extremities (13.9%) [3]. Adenocarcinoma of sweat glands appears as moderate to poor adenocarcinoma with regional variations, ranging from true ductile form to infiltrative anaplastic form. Histologic view of malignancy is similar to most epithelial tumors. The distinction between metastatic adenocarcinoma and primary adenocarcinoma of the sweat glands can be challenging [4].

## Case presentation

A 37-year-old woman presented with a painless nodule in her umbilicus which histopathology examination suggested to be a malignant umbilical tumor. Fourteen months before admission, the patient had a history of umbilical tumor surgery, with histopathology examination suggesting moderately-differentiated adenocarcinoma. The patient also had additional oral chemotherapy six times, using Capecitabine 2 × 1500 mg. The patient complained about a recurrent mass in her umbilicus at the surgical scar site.

**Fig. 1:**
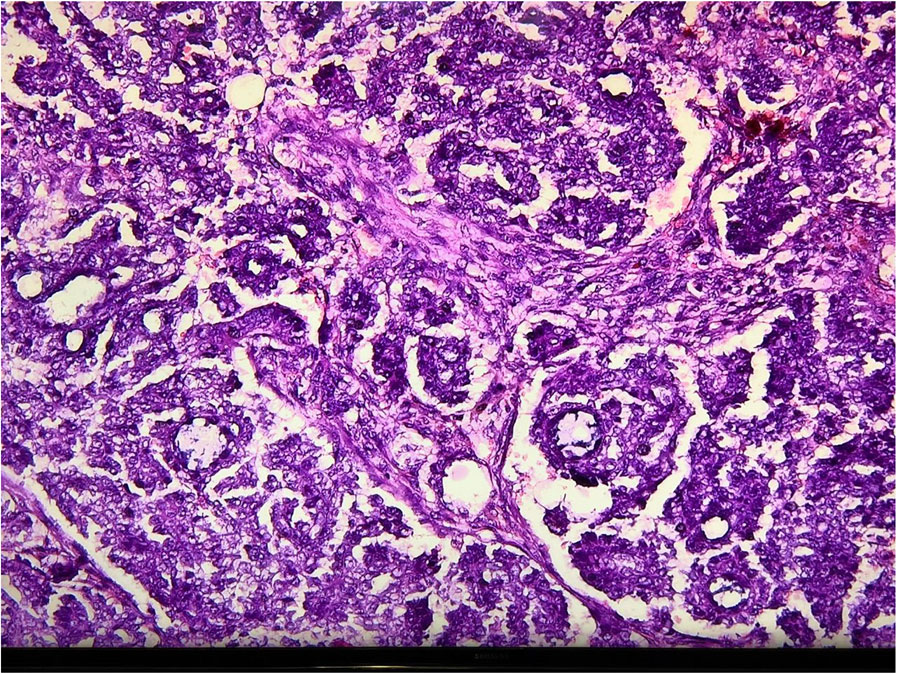
Pathological anatomy view at 100× magnification

**Fig. 2:**
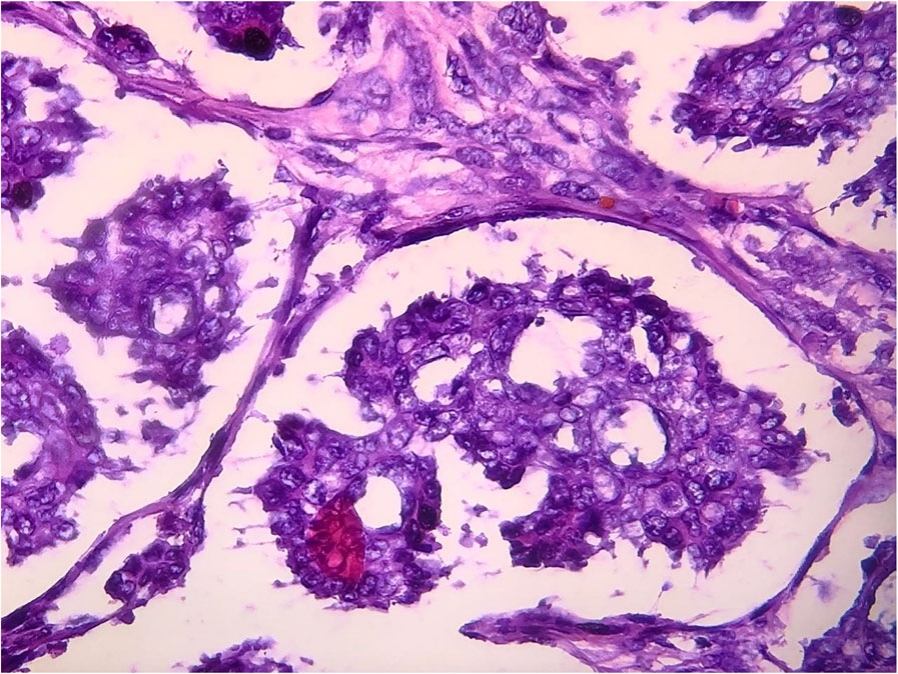
Pathological anatomy view at 200× magnification

On examination, cytology examination using Fine Needle Aspiration Biopsy (FNAB) results identified some malignant cells (+). As seen in Figs. 1 and 2, the adenocarcinoma of the sudoriferous gland is arranged into tubular and papillary patterns consisting of polymorphic cells, scanty cytoplasm, irregular nuclei, and coarse chromatin. Colonoscopy examination was performed to ascertain whether the tumor was primary or secondary colonic metastasis. Results were in the normal range, without intraluminal mass or stricture, and subsequent colon mucosa biopsy showed chronic colitis. CT (Computed tomography) scan was also performed, and the results showed no metastasis.

**Fig. 3:**
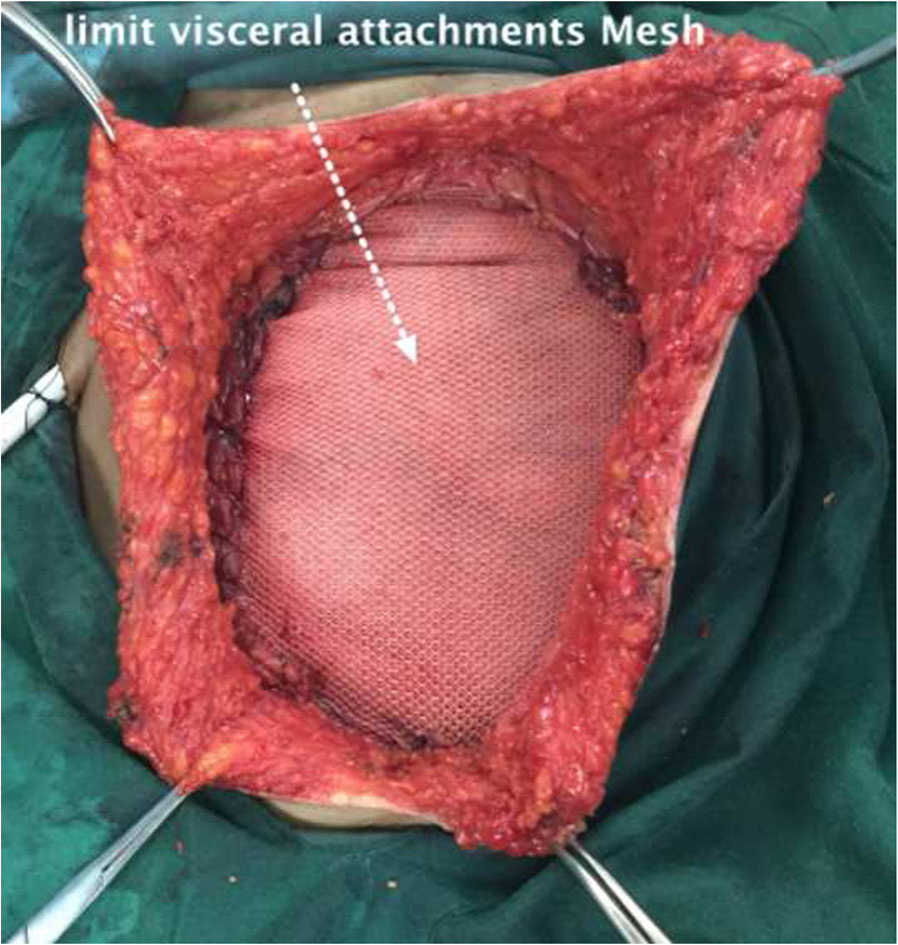
Wide excision dan abdominal reconstruction using mesh

**Fig. 4:**
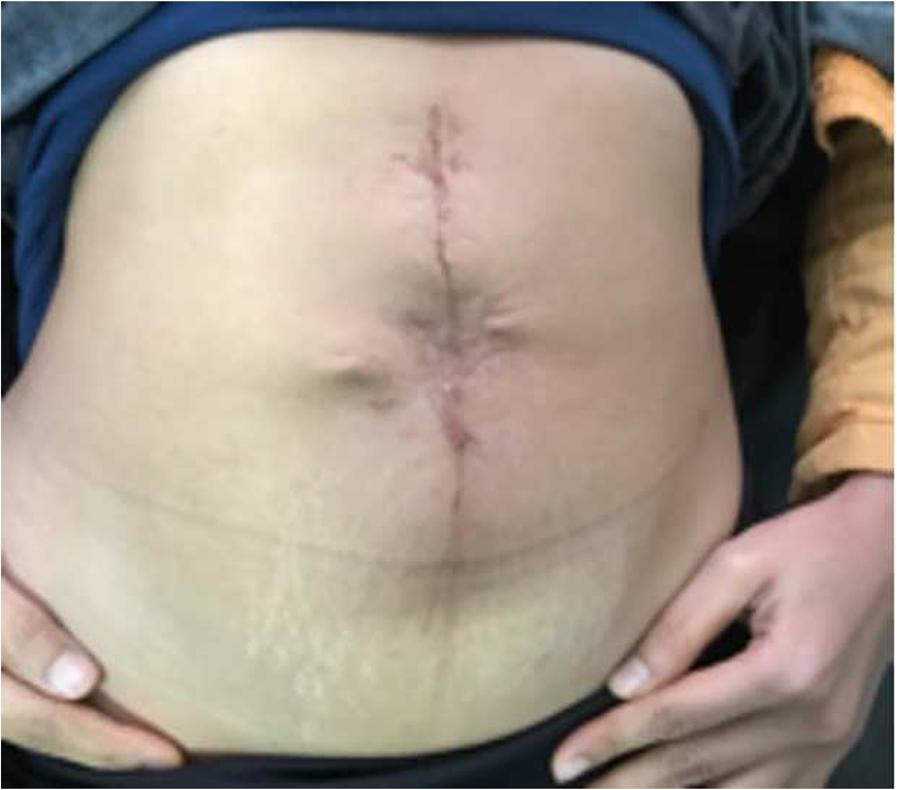
Two years after surgery

A recurrent tumor mass of adenocarcinoma with the diameter of 7 cm had been excised with the tumor margin of 5 cm. Wide excision surgery was performed leaving a 17 cm surgical defect on the anterior abdominal wall (Fig. 3). The reconstruction was performed using anti-adhesive *Parietex polyester* mesh. Reasonable collagen barrier on one side to limit visceral attachment was sized 30 × 30 cm. Histopathology examination of the excised tissue suggested sudoriferous gland adenocarcinoma with adjacent tissue free of tumor cells. Treatment was continued with additional chemotherapy using Capecitabine 500 mg dose 3–0-2 mg and Bevacizumab (Avastin) 400 mg 12 times. Follow up PET (Positron Emission Tomography) scan six months post-surgery was performed and showed no residual tumor in the umbilical region, and no apparent paraaortic nor mesenteric lymphadenopathy. Postoperative follow-up after 2 years is shown in Fig. 4.

## Wide excision and reconstructive surgical technique

- Surgical technique decisions were based on pathologic review from prior umbilical tumor resections
- Our goal was excision of the tumor with negative gross and microscopic margins of resection after the previous recurrence.
- During surgery, we attempted to achieve a gross margin after recurrence with the excision distance from the grossly palpable lesion greater than 5 cm, while normally in first surgery we can performed with the margin 1–2 cm.
- We performed full-thickness resection of the tumor-containing abdominal wall continued with exploratory laparotomy to evaluate the intraperitoneal extent of the umbilical tumor.
- Residual defect of abdominal wall reconstruction was performed using prosthetic mesh
- The mesh in a tension-free manner was sutured with combined multiple anchors and continuous running suture to the edges of the fascial defect.
- Wound closure was performed following placement of subcutaneous vacuum drainage.

## Discussion and Conclusions

Umbilical cancer has a reasonably high incidence, about 10% of all malignant tumors present on the abdominal wall. Most of umbilical tumors (80%) are from visceral organ metastases (Sister Mary Joseph Nodule) because they have adjacent vascularization and embryonic connections, while the remainder are primary tumors [1]. In our case, as in the case of primary tumors, no primary tumor was found based on colonoscopy and CT scan. Clinical symptoms that can arise include pain, ulceration and necrosis, and sometimes it can also discharge mucous or blood. Other signs such as irregular bumps can appear that enlarge progressively ranging in size from 0.5–2 cm [2, 5, 6].

The diagnostic tests used to diagnose this tumor are usually using radiologic modalities, such as cystogram, ultrasonography (USG), magnetic resonance imaging (MRI), computed tomography scan (CT-scan), and positron emission tomography (PET). Abdominal sonography usually produces the normal result [7]. In some cases, an irregular hypoechoic mass can be found with microcalcification in the umbilical region, but not extending into the peritoneal cavity [1]. Doppler imaging sometimes can show internal vascularity [8]. Local staging and evaluation of distant metastases of the tumor often uses CT scan and MRI. On the CT scan images, the tumor appears as a mixed solid and cystic mass. Differential diagnosis of the umbilical nodule includes neoplastic or non-neoplastic lesions such as Paget’s disease, angioma, umbilical adenoma (raspberry tumor), umbilical hernia, endometriosis, hypertrophic scar, and umbilical granuloma. Tissue diagnosis using fine-needle aspiration biopsy (FNAB) is acceptable to establish the diagnosis [9].

The recommendation of management of primary umbilical tumors after the excisional biopsy is extensive surgery with a 1–2 cm tumor-free margin along with lymph node removal when clinically positive. A broader excision may prevent recurrence [3, 7]. The main reason we did surgery with a 5.0 cm margin was that our case is a recurrent sweat gland umbilical adenocarcinoma, and needed a wider excision to prevent its recurrence. In our case, we also used an anti-adhesive mesh, and neither a rejection nor a postoperative hernia occurred.

In surgical management, the greatest difficulty lies in the reconstruction of the umbilicus because its depth should be sufficient, especially when the patient presents a scarce panniculus adipose. By ensuring an adjacent fatty tissue is included, this technique solves this problem. Maintaining a curve in the base of the flap rather than a right angle to the approach avoids the formation of edges, thereby achieving a more rounded umbilicus and avoiding one that is elongated and closed [10]. Several studies recommend adjuvant chemotherapy and local radiotherapy as well as lymph node dissection as prophylaxis in highly undifferentiated tumors. The cutaneous sweat gland carcinomas are radioresistant, and chemotherapy has been infrequently employed.

In some cases, a more aggressive approach is used, because when metastasis occurs then the prognosis will worsen. Primary adenocarcinoma of the umbilicus from cutaneous sudoriferous glands is an uncommon neoplasm that may behave more aggressively than most tumors. Management of primary umbilical tumor involves surgical excision. Radical local umbilical surgery and reconstruction with limit visceral attachments is indicated [1]. In conclusion, wide excision and reconstruction surgery for recurrent sweat gland umbilical adenocarcinoma followed by chemotherapy can be an alternative to prevent recurrences.

## Abbreviations

CT: Computed tomography
FNAB: Fine needle aspiration biopsy
MRI: Magnetic resonance imaging
PET: Positron emission tomography
USG: Ultrasonography
